# “I Spent a Full Month Bleeding, I Thought I Was Going to Die…” A Qualitative Study of Experiences of Women Using Modern Contraception in Wakiso District, Uganda

**DOI:** 10.1371/journal.pone.0141998

**Published:** 2015-11-02

**Authors:** Simon P. S. Kibira, Christine Muhumuza, Justine N. Bukenya, Lynn M. Atuyambe

**Affiliations:** 1 Department of Community Health and Behavioural Sciences, School of Public Health, College of Health Sciences, Makerere University, Kampala, Uganda; 2 Centre for International Health, Department of Global Public Health and Primary care, Faculty of Medicine and Dentistry, University of Bergen, Bergen, Norway; 3 Department of Epidemiology and Biostatistics, School of Public Health, College of Health Sciences, Makerere University, Kampala, Uganda; the University of Sydney, AUSTRALIA

## Abstract

**Introduction:**

There is high unmet need for family planning (FP) in Uganda as well as high contraceptive discontinuation rates. These contribute to the high fertility rates that in part are due to unplanned pregnancies. There are gaps in knowledge about experiences that couples go through while using contraceptives in their lives. This study explored women’s experiences during the course of their contraceptive use.

**Methods:**

We conducted a qualitative study involving 30 women who had used modern contraception for at least one year in Wakiso district, central Uganda. We used in-depth interviews to obtain their personal accounts. Index women were approached through health officers at four health centres in the district. All ethical approvals and informed consent were obtained. We used conventional content analysis; identifying codes through open coding, on which basis categories were developed and grouped into overarching themes.

**Results:**

Women’s accounts were summarised in the following themes: negative experiences with modern contraceptive use, motivation to continue using FP in spite of these negative experiences, the role of influential people, and discontinuation of use. Negative accounts dominated the experiences of most women but they expressed strong desire to continue using modern contraception even amidst all challenges. Health workers emerged as the most influential people that played a vital role in women’s decisions.

**Conclusion:**

Varied negative experiences with modern contraception and misperceptions exist amidst a determination to continue use. Partner engagement, health service strengthening to improve side effects management and health worker skills, and engaging older women that have successfully used contraception as community champions, are potential strategies to support women’s contraceptive decisions.

## Introduction

Women have control over their own fertility only when they are able to decide if, when, and with whom to bear children. This implies the right of women to accurate information on family planning (FP) in addition to safe, effective, affordable, acceptable and legal services of their choice [1]. An increase in modern contraceptive use is vital if women are to control their fertility ambitions. However, modern contraceptive use is still low in most sub Saharan African countries including Uganda where population growth, fertility and unmet need for FP are high [2]. Uganda is projected to have an over five-fold increase in population by 2100, and is among the top six of the eight countries that will account for more than half of the world’s projected population increase[3].

There has been a significant reduction in fertility the world over [4], but in Uganda this decline has not been realized. The total fertility rate in Uganda remains high [6.2 in 2011 [5] down from only 6.9 in 2001 [6]], and is due in part to low contraceptive use. The latest National health indicators show that only 26% of married women use modern contraceptives with 33% expressing an unmet need for FP, even when knowledge of at least one method is nearly universal (97%) [5].

There are fears and side effects that bar some women from uptake and continued contraceptive use [7]. Results from the two most recent national demographic and health surveys in Uganda [5, 6] and a sub-Saharan African study [8] attribute the unmet demand in part to fear of side effects of modern FP methods and opposition of influential people in women’s lives, such as male partners. Fear of side effects is also the most commonly cited reason for four out of ten Ugandan women discontinuing use of FP within 12 months of initiation.[5]. Specific concerns about different modern FP methods including weight gain, amenorrhoea, irregular and heavy bleeding, unwanted vaginal wetness and dryness, pain and lower libido among others have been documented among both first time and continuing users [9–14]. Indirect method related factors such as lack of partner support [12] and money to purchase contraceptives have also been reported to contribute to contraceptive discontinuation [15].

Family planning methods use is interdependent on the demand and supply side as well as barriers in between [16]. FP service provision in Uganda has limitations in quality [17] arising from several factors. For example, the health worker limited skills set [16, 18, 19] to comprehensively provide all the methods at the various health facility levels as well as the attitudes of some providers towards provision of services to select groups like adolescent women [18] can be barriers. Health workers may also have their own perceptions [18, 19] towards certain methods that can influence service provision and method choice for women and couples. Yet, health workers are vital to women’s contraceptive use decision making [20, 21].

There are few qualitative studies that focus on users experiences of modern contraceptives [14, 22]. Many quantitative studies report side effects as a barrier to FP use and report contraceptive discontinuation rates [5, 6] without reasons for such discontinuation. This study explored lived experiences among female users of modern contraceptives, their challenges and motivation for continuation. This could inform the provision of acceptable rights-based FP services.

## Methods

### Setting

This study was conducted in Wakiso district, which is in central Uganda with a population of two million people, 53% of whom are women and 68% rural according to Uganda Bureau of Statistics projections [23]. The district is home to the largest urban population outside the capital city, and also has the most populous rural sub-county in the country, making it ideal for the study due to this unique rural-urban mix. The study was conducted in six sub-counties served by five level four health centres (HC IV). The district has 103 facilities including 4 hospitals, 5 Health Centre (HC) IVs, 37 HCIII and 57 HCIIs. A HC IV is one level lower in hierarchy to a hospital, should serve a catchment population of 100,000 and offers inpatient, outpatient, reproductive health and surgical services. Among critical technical staff, it should be staffed with a medical doctor, at least two clinical officers, one senior nursing officer, three midwives, and four nurses.

### Study Design

Thirty in-depth interviews were conducted between August and September 2013 with women who had used modern contraception for at least one year. In depth interviews were the best suited to explore barriers and facilitators of continued FP use, and capture lived experiences in detail. We used the “dhs program” categorisations of modern methods of contraception in our in depth interview guides; female and male sterilization, the oral contraceptive pill (pills), IUDs, injectables, implants, male and female condoms and emergency contraception [5].

### Study informants

We explained the study purpose and procedures to the health workers in charge of FP services at each of the four health centre IVs that were selected. Health workers referred us to women clients that had used modern contraceptives for at least one year even if they had stopped using at the time of the study (this was the inclusion criteria). They were contacted through their mobile phones numbers after initial notification from the health worker(s). The women identified then acted as index informants, leading us to other users living in the same community within the district in a snow ball approach (Table 1). All 30 women approached agreed to participate in the study.

**Table 1:** Number of participants and how they were approached.

Name of Facility	Identified through Health workers	Approached through fellow women	Total women from each facility
Wakiso HCIV	3	5	11
Kasangati HCIV	4	9	13
Ndejje HCIV	2	1	3
Namayumba HCIV	1	2	3
**Total**	**10**	**17**	**30**

### Data Collection

We recruited two experienced research assistants competent in the local language and trained them for two days on the study protocol and procedures. The tools were translated and pretested prior to data collection. Interviews were conducted by the two experienced research assistants (one with a Master’s degree and another with Bachelor’s degree qualification) and one of the authors (CM) with knowledge of the local language. The women were interviewed at their homes.

The interview guide focused on areas such as: the methods of contraception currently used; for how long women had used methods and experiences though the periods of use; how well they liked the family planning methods that they were using (probing for both the good and less liked sides); how women coped with the challenges they reported; how they accessed particular methods; plus who and how supportive the influential people in their lives were. We also asked about the modern methods used in the past but had been stopped and circumstances under which they had discontinued use.

All the interviews were conducted in Luganda, the local language that respondents were comfortable with. Interviews were audio recorded, simultaneously transcribed and translated to English. They lasted about 40 minutes. Although we could have reached data saturation with fewer interviews [24], we interviewed 30 women to have a “confirmation” of varied experiences reported in the first few interviews.

### Data Analysis

We used conventional content analysis [25] with the codes and categories arising from the data. Transcripts were read several times to get an overall impression of the data, and identify initial codes, noting repeated issues and emerging ones on which categories were developed. The categories were grouped together into overarching themes based on the authors’ understanding of the data. Transcripts were manually coded by the first two authors (SPSK and CM) overseen by the last author (LA) who also checked a sample of the coded transcripts for consistency.

### Ethical Considerations

We obtained ethics approval from Makerere University School of Public Health Higher Degrees Research and Ethics Committee (IRB 00011353) and the Uganda National Council for Science and Technology (SS 3217). We also obtained permission from Wakiso district health office and from health officers that were in-charge health of each of the six health centres. Study details were well explained to all participants and written informed consent obtained. Privacy was ensured in all the 30 interviews and data was kept confidentially with access restricted to only the investigators and the two research assistants.

## Results

The 30 informants were women aged 20 to 45 years who had used modern contraceptive methods for periods ranging from 18 months to 18 years and who were using either oral contraceptive pills, contraceptive injection (DMPA), IUDs, implants or male condoms at the time of the interview. Four women (three living in urban and one in rural area) had stopped modern methods at the time of interview after using them for three to ten years. Their age at initiation of modern contraceptive use ranged from 17 to 34 years. Thirty three percent (10) of the women were identified through the health worker references, while the other proportion was through snowball method. Most women were linked by the index identified from Wakiso and Kasangati HCIVs (Table 1).

Four main themes emerged during analysis as indicated in the coding framework (Table 2). These were: negative experiences with modern contraceptive use; motivation to continue using FP in spite of the negative experiences; the role of influential people; and discontinuation of use.

**Table 2:** Example of the coding Framework used to arrive at themes.

Theme	Categories	Examples of Codes
Negative experiences with modern contraception	Physical experiences	Weight gain, Reduced libido, heavy bleeding during menses, Weight loss, Prolonged menses, Nausea, Vaginal dryness.
	Psychological experiences	Living with unsupportive partners, Fear of infidelity from partners because of side effects, Inconvenience from methods use such as the worry of remembering to take the daily pill.
	Financial experiences	Costs of methods, costs of treating side effects, purchase of extra sanitary pads in case of prolonged bleeding, Loss of productive work time due to side effects.
Motivation to continue using contraception	Financial benefits	Indirect financial benefits arising from having smaller families
	Psychological satisfaction	Less worry for unwanted pregnancies and children, having healthy families
Influential People	Health workers	Counselling women on alternative methods, counselling on method choice for initial use, advice on managing side effects and enabling coping, confidants especially in case of stealth use
	Partners/ husbands	Providing transport to facilities, allowing women to use methods, finances to buy methods, financing the treatment of side effects, reminding women to take pills and go for refills, discussing family planning with women.Negatively responding to side effects, opposing use of methods.
	Close female friends	Sharing positive experiences from past use, sharing negative experiences, spreading rumours about myths,
	Older women in the communities	Agitating for more children for women, spreading myths
Discontinued used	Misperceptions	Causing cancer, causing fibroids, cause infertility, foetal mortality
	Side effects	
		Inconvenience
	Partner opposition	Want more children, cannot bear with side effects
	Switched to other methods	To other modern methods, to folk methods, no methods

### Theme I: Negative experiences with modern contraceptive use

All the 30 women reported negative physical, psychological and financial experiences with FP methods particularly at the initial stages of use. Negative experiences were most cited for hormonal methods (especially the injection and the Implants), while minor inconveniences like discomfort and reduced vaginal lubrication were more reported in connection with non-hormonal methods; male condoms and IUDs. Women experienced a mix of several physical side effects ranging from gaining unwanted weight for some women, or losing weight, over bleeding, extended menstruation days, delayed menses, reduced libido, reduced lubrication during sexual intercourse among others. These experiences varied for different women.

The typical quotations below highlight some experience for women using the injection and the implants.

A woman using injection method narrated:

*…I gained weight*, *then after I stopped going into my periods*. *It took me six months to start noticing weight gain*… *It also took me a full year without going in my periods and when I resumed*, *I had heavy bleeding*. *The biggest burden was heavy bleeding and the days of my menstrual cycle had increased from four to a whole week or one and a half weeks at times*. (38 year old)

In the same vein, another young woman who was still using implants at the time of the interview noted:

*At the first time*, *I thought I was going to die*. *I spent a full month bleeding*. *I thought I was going die because whenever I was standing*, *I would feel my legs shaking*. *My husband was concerned and insisted that we should go and see the doctor*. *If you do not have money*, *do you see how you die*? (23 year old)

One woman who had switched from injectables to occasional condom use said:

*You know that injection [DMPA]*, *it reduces your libido and in the event that you have sex with your husband*, *you just force it*. *You do not enjoy and at times it is very dry [not lubricated]*. *If you add the injection to the general mood swings you totally lose sexual appetite*. *I used to notice it*. *I do not know whether even those who use pills and implants feel the same*. (40 year old)

In addition to physical side effects, psychological challenges cited included the inconvenience of FP use, living with unsupportive partners, and worry that the experience of side effects would lead to partner infidelity in the event that they did not have the sexual satisfaction from their relationships.

Women also cited financial challenges that included the costs of treatment for side effects, costs of extra sanitary pads and productive time lost due to side effects.

*They [health workers] examine you and tell you that ‘you have this problem*, *but we do not have treatment for it*.*’ The medicine [for treating side effects] that we buy in clinics should also be here in [public] health centres*. *Sometimes you come without money*. *Me I had some money but there are times when you do not have even 100 Uganda shillings [about 30 US cents] but you are bleeding*.(23 year old).

The other concerns were the inconvenience with using the pills due to daily intake. For two women, the pill burden was reportedly very challenging that it resulted in non-adherence and unwanted pregnancies.

### Theme II: Motivation to continue using FP even with negative experiences

All 26 women who were still using modern contraceptives were motivated to persist by indirect financial benefits resulting from smaller manageable families, psychological satisfaction with less worry about unplanned pregnancies, and having healthy and well-spaced children.

*…I have only had prolonged monthly periods and I delayed going into my periods for like 3 months*. *The Injection has helped me to have my children grow well*. *If I was not using it*, *I think I would produce every year*, *but it has helped me to space them as I wanted*. *Those are the good things that I have received from using the injectaplan*. (26 year old)

Others did not want to have any more children after realising the burden of caring for those they already had. This young woman’s quotation shows the typical responses for limiters.

..*Even when I got complications I could continue using [implants] because I never wanted to produce more children*. *I already have enough children [had 3 children at age 20 when she started using]*, *yet I am still young*. (25 year old)

### Theme III: The role of influential people

Women relied on health workers, husbands, close female friends and older women in the community as influential people in making decisions about method choice, switching or coping with negative experiences.

Nearly all women cited the expert professional advice provided by health workers as the most significant in shaping contraceptive use decisions. They gave advice on use of certain methods and counselled women about possible side effects. This quote illustrates how strong health worker advice was regarded:

*The implants were recommended to me by the health worker even though I knew a neighbour who was using them*. *But that woman had suffered with them so much and they were removed*. *But for my case*, *the health worker told me it would match with my body since I had used the injectables before*. *So I followed her recommendation to use it*. *And now I am using implants*. (25 year old)

Health workers were also supportive in enabling women cope with side effects by providing medications, counselling and advice on alternative methods. Trust was vital in the relationship between women and health workers particularly for young women who were using FP without their partners’ knowledge.

*I always talk to the health provider and she finds a way of helping me*. *I cannot take a decision on my own regarding these challenges*. *When I got the injection and experienced problems*, *I came back and talked to her*. *She told me ‘such things happen at the start but you will be fine after some time’*, *and I indeed I got well after*. (26 year old)

*I did not tell anyone about my side effects with implants [stopped using it] except the health providers because people can spread rumours and yet my husband does not want me to use it*. (25 year old)

The role of marriage partners was both supportive and negative. Seven women noted that their partners provided ‘permission’ to use FP, provided finances for transport to the facilities, and for purchasing contraceptives and FP-related health care like treating side effects, as well reminding partners to take the daily pill.

*…my husband is very supportive regarding family planning*. *We always sit with him and discuss planning for our family*… *He always ensures that I do not forget the period to go back for refills and reviews*… *I think because he also sees that the children we have are enough*. (38 year old)

However, other women reported lack of support from their partners due to desire for more children, or in genuine response to side effects affecting their wives, notably reduced libido.

*I have not told my husband that I have bought the tablets again [to help cope with the heavy bleeding from Implants]*. *The first time he is the one who bought them because the doctor asked him to buy them and he thought that I got well*. *[But] now I went and bought them myself because if I had told him*, *he would have asked me to stop using contraception*. *So I kept it to myself*… (23 year old)

Close female friends were supportive if they had positive experiences with FP themselves. Such friends played the role of ‘experts’ in helping peers to cope with negative experiences. However, friends with negative first-hand or rumoured experiences discouraged women from using contraceptives. Often the rumoured experiences reported turned out to be misperceptions as noted in the following quotes from a young and an older woman.

*They [other women friends] were telling me that the IUD changes location once inserted and goes deep into the body of the uterus and causes cancer*. *As a human being I am scared*. *Women talk a lot here*. *My friends discourage me because they are not using family planning*. *So when I hear them say this*, *it makes me feel bad yet I do not want to follow their negative talk*. (22 year old)

*They say that there are many cases of cancer in Mulago [national referral] hospital due to the IUD*. *So we get scared and we have failed to make choice of what method to always use*. (40 year old)

Likewise, some older women in the community were a negative influence on younger women, urging them to have more children and not use contraception.

### Theme IV: Discontinuation of use

Some women expressed inability to handle the challenges from specific methods and decided it was time to give up completely or switch to other methods that they hoped had fewer challenges. We have categorised the reasons given for termination of methods into: misperceptions about methods, side effects, inconvenience and partners’ unwillingness to use. Misperceptions were mainly from the community and close friends and included: infertility when taken over prolonged periods, foetal death in case one conceived while on contraception, and uterine fibroids and cancers. One woman for example diagnosed with fibroids said this must have resulted from a prolonged lack of menstrual blood that was retained intra-uterine due to use of injectable contraception. Women believed that over time hormonal contraception as well as the IUD destroyed reproductive functioning. A woman who had used pills perceived the death of her foetus to have resulted from pills.

*I got pregnant [while taking pills] and the foetus was damaged because I continued taking them [without knowing I was pregnant]*. *They had to wash my uterus because it was a pre-mature*. *It was so painful*… *I was breastfeeding and at the same time I was taking some pills and that time I was feeling a lot of pain in the stomach and I was crying all night*. *So when we went to Mulago [national referral hospital] for a check-up*, *they told me I had a dead foetus*. (40 year old)

Another woman also had similar myths for the pills.

*I stopped pills*… *I had taken them for three years but people say when you swallow them they settle in one specific place in the stomach*. *Just imagine how big the ball in my stomach would be by now*! *I also hear they cause fibroids*, *so I had to change*. (35 year old)

Of the four women who had discontinued use of modern methods completely, two opted for withdrawal and rhythm methods; one did nothing while hoping she would not get pregnant; and the other used folk methods

*After stopping using pills*, *I used some local methods*… *It was a thread tied round my waistline but when I lost that thread*, *I got pregnant*. (40 year old)

## Discussion

This study has attempted to explore women’s experiences of using modern contraceptives. We summarise women’s accounts in four thematic areas: negative experiences with modern contraceptive use, motivation to continue using FP amidst negative experiences, the role of influential people; and discontinuation of use. Negative accounts dominated women’s experiences in this study and were the main reason for discontinuation of contraceptive use for the women that had stopped use, as well as involuntary switching of methods. Women were nevertheless motivated by the lure of smaller planned families and psychological benefits, to continue using contraception with the support of health workers, friends and their male partners. The negative influence of unsatisfied users, some older women and some unsupportive partners were deterrent to continued use of FP.

Negative experiences with modern contraception complicate continued use of contraceptives particularly when partner support is limited. Surveys in Uganda consistently show that fear of side effects is the leading reason for non-use of contraception even among women with an expressed need [5, 6, 8]. The accounts in this study confirm that there are genuine side effects that women face [26], as well as misperceptions that negatively affect continued use. The common side effects of modern contraceptives have been reported among women in varied settings [7, 14, 22, 26–28]. This study presents detailed accounts of women about these effects, complementing accounts from similar research [14, 29]. This implies that FP service providers need to actively engage women into discussion of side effects and offer solutions that could include switching to less troublesome methods for different women. As noted by Higgins and others, methods that induce more side effects are less attractive [30, 31].

Misperceptions about specific methods and FP in general were widely reported in this study. Allaying fears from myths coming from the communities and close friends could depend on the information that women are equipped with from health facilities during counselling and health education [32, 33], as well from social marketing campaigns for FP. Similar misperceptions such as cancer and infertility have also been reported among postpartum women in Uganda [12] and among women and men in urban Kenya [26]. Such misinformation needs specific attention [7] to tackle involuntary discontinuation and potential barriers to uptake of contraception.

The pattern in the data indicates that the major negative experiences as well as misperceptions in this study were most commonly cited for two hormonal methods; injection [14] and the Implants. Women seem to report more concerns for these two methods yet they were also reportedly the most used. Plausible explanations for injectable preference could be: the long term nature of the methods, ability to control the method without the opposing partner’s knowledge, convenience [14, 22], as well as method availability for the injectable at public facilities in Uganda. There were relatively minor inconveniences for non-hormonal methods in the study. Further promotion of the entire range of varied alternatives as well as provision of such at public facilities at the lower levels could be a solution for women that want to switch methods.

This study reveals the financial implications of side effects that have also been reported elsewhere [26, 34]. Given the inadequacies in the public health system [18, 35], FP users are likely to seek treatment for side effects from private health facilities which may not be affordable [36] to the majority. It is important that in the interest of equitable rights-based services, provision of free public sector FP services is accompanied by readily available remedies for side effects. Worth noting is the indirect financial benefit from smaller families that women reported to be associated with using FP methods [12, 26]. This is already part of the FP social marketing and should be a main stay of such interventions. The immediate negative financial implications may overshadow this long term benefit if there is a lapse in counselling. Further research to understand the financial impact of FP side effects is recommended.

The rationale for complementary free, available and acceptable services for the management of side effects is strengthened by the strong motivation shown by women in this study to continue using FP in spite of pervasive negative experiences. Partner support emerges as a driver of motivation [21, 26], underscoring the need for male partner engagement on FP discourse and has been recommended [37, 38]. Some women have reported inability to make contraceptive use decisions without partners who are easily influence by the negative information [12]. Partners in our study were also reported to negatively influence contraceptive use especially when they feared side effects for their loved ones and the financial and psychological consequences of bearing with these effects. This has been reported in other studies [14].

Having healthy, planned, well-spaced pregnancies and children [12, 20] emerged as another powerful driver of persistence with contraception in this study. These indicate the further need for male engagement [38] and children’s wellbeing as critical elements that FP promotion programs should continue emphasising in motivating women to sustain contraceptive use.

Key influential people in women’s contraceptive use journey are another element that current FP promotion and service delivery services may not be emphasizing, but who emerge as important in this study. Health workers were trusted powerful influencers in use and continuation decisions for women, and have been reported to influence contraceptive decisions [21, 26]. They therefore need to be well equipped with information and skills [39] to fulfil their ‘expert’ role which sometimes is subjected to own mixed perceptions [18] and has limitations [16, 19, 20, 39]. Maintaining FP user trust in health workers through skills enhancement and provision of interpersonal skills is essential for FP use and continuation decisions.

The negative influence of some older women on contraception continuation was widely noted by our participants, suggesting that older women are opinion leaders in this area. Traditional demand generation strategies have employed the use of among others, positive deviants [40] and satisfied users in promoting behaviour change. Contraceptive use could benefit from such interventions that have also been recommended before [17]. Engaging older women that have successfully used contraception as FP champions is a strategy that is not currently widely exploited but has potential for motivating younger women. The importance of trusted, community based champions is underscored by the finding that women discontinued contraceptive use on the basis of misconceptions [12, 41], resorting to unreliable folk and traditional methods.

One of the strengths of our study is that it explored lived experiences of FP use among women with varying socio-demographic profiles, who had used contraception for varying durations. However, due to the small sample of women in one district of central Uganda, our findings are not generalizable but we believe can be transferred in similar settings in Uganda. By design this study is best done with such small numbers to give a deeper understanding of experiences of individual women. This study was only conducted among long term users and excluded users who had used methods for less than 12 months because in our design we expected these women to have less varied experiences. We acknowledge that personal experiences of naive users may be slightly different from experienced users and women may be more likely to remember negative than positive events. These results need to be interpreted in the context of these limitations.

## Conclusions

Pervasive negative experiences with contraception exist amidst a determination to continue use of contraception among study participants. Partner engagement, health service strengthening to improve side effects management and health worker skills; and engaging older women who have successfully used contraception as community champions are potential strategies that can be employed to support women’s FP use decisions. The important role played by male partners should also be promoted by FP programs. Indirect contraceptive benefits that clients may see as opportunity costs to encourage them to overlook the contraceptive side effects and their negative financial implications should be enhanced in the social marketing of FP.

## Supporting Information

S1 Text Interview guide. (PDF)

S2 Text Excerpts from interviews. (DOCX)
