# Supplementary material for: “I Spent a Full Month Bleeding, I Thought I Was Going to Die…” A Qualitative Study of Experiences of Women Using Modern Contraception in Wakiso District, Uganda
Source: PLoS One. 2015 Nov 2;10(11):e0141998. doi: 10.1371/journal.pone.0141998 (PMC4629884; doi:10.1371/journal.pone.0141998)
Supplement: S1 Text — (PDF) [file pone.0141998.s001.pdf]

Example of the Interview guide used

1. What is your opinion of the family planning services provided at your health facility? *(probe for how they like the services, the health workers attitudes, costs)*
2. What are the most popular modern methods of contraception that are used by individuals and couples in this community?
3. Now, let us talk about your own life. What method(s) of contraception are you currently using? *(Probe for reasons for choice of those methods, if this was the first method used)*
4. For how long have you used this (these) method(s)? *(probe for when, at what age they started using FP, any discontinuations in between, any change of methods)*
5. How do you like the family planning method(s) that you are using? *(probe for the good and bad, side effects)*
6. How do you cope with the challenges in using modern family planning methods?
7. How do you access these methods? *(probe for sources, ease of access, costs of methods, period to refill, stock outs)*
8. Let us talk about the people in your life, how supportive are they about using these method(s) of family planning? *(probe for significant others support or discouragements, who is supportive and non-supportive, how she feels about these people and their views)*

9. Have you used any modern methods in the past but have since stopped using them? IF No why. IF YES, tell me about those method(s)? *(Probes: what were they, where did you obtain them from, how did you obtain them, who recommended them)*
10. For how long did you use the methods? *(probe for period of use and when they stopped using the method)*

11. Under what circumstances did you to discontinue the use of the method(s)? *(probe for access issues; costs of methods; side effects; who influenced decision:- providers, peers, significant others)*
12. *If currently not on any modern methods of FP*, are you doing anything /using any means to stop getting pregnant/ making your partner pregnant? What are you using?
    If currently not on any modern methods of FP, (probe for what they are doing in detail)
13. What would you want to tell other women/ men who have not started using contraception or those who have discontinued but are sexually active?
14. Do you have anything else you would like to share with us about modern family planning methods?
