# Supplementary material for: “I Spent a Full Month Bleeding, I Thought I Was Going to Die…” A Qualitative Study of Experiences of Women Using Modern Contraception in Wakiso District, Uganda
Source: PLoS One. 2015 Nov 2;10(11):e0141998. doi: 10.1371/journal.pone.0141998 (PMC4629884; doi:10.1371/journal.pone.0141998)
Supplement: S2 Text — (DOCX) [file pone.0141998.s002.docx]

# Relevant Excerpts from Interviews

**Source Transcript: IDI – FP – KS – TR – 04 (38 years)**

Interview (I): Basing on your seven years’ experience on injectables, what do you have to tell me about this method?

Respondent (R): I got challenges with the injectables because before I used this method I was a small lady but when I started using injectables I gained weight, then after I stopped going into my periods. It took me six months to start noticing weight gain... It also took me a full year without going in my periods and when I resumed, I had heavy bleeding. The biggest burden was heavy bleeding and the days of my menstrual cycle had increased from four to a whole week or one and a half weeks at times

………………………………………………………………

I: Let us talk about the people who have meaning in your life, how positive are they about using these methods of family planning?

R: People like my husband; especially my husband is very supportive regarding family planning. We always sit with him and discuss planning for our family...

I: How does he support you?

He always ensures that I do not forget the period to go back for refills and reviews. And he always advises me not to withdraw from use of family planning. I think because he also sees that the children we have are enough

I: What are some of discouragement that have come your way in regard to the use of family planning use?

R: Especially our friends, you may find a friend who will tell you that if you go for family planning you are looking for illness for nothing, you are looking for illness for nothing, you will die for nothing, family planning causes infections in women, so they always discourage as friends.

**Source Transcript file name: IDI_FP_NB _02_CM (23 years)**

I. now madam, as we are about to end, is there anything else you would wish to tell me or to share with me concerning family planning that we didn’t talk about here?

R. I just want to request you, now that you decided to help us, help us fully. Also these tablets that we buy in clinics, if you brought them in health centres and make them available so that when someone got a problem, she just comes like a malaria patient because even if you came here, you won’t get it. They only prescribe for you and you buy from the clinic because after buying and swallowing it, you are like any other normal person. Now what I am scared of is, taking a lot of it also causes problems.

I. I beg your pardon?

R. what I said is that when you take a lot of it, it brings you problems which I don’t know.

I. it brings you problems?

R. the problem is that I take them after every 1 month; they might also cause me problems. That’s why I am saying if they helped us and put it in the health centre so that if a person came and is having a problem with family planning, it is given to her.

I. for free?

R. yes for free.

I. so the problem that you have is that even if you used family planning and you got a problem, you have to buy it?

R. yes

I. and sometimes…

R. and there are some clinics that sell it expensively, there are times when it’s scarce and they are selling 2 tablets at 5000 shillings. There are 2 tablets which you take at once and they sell them at 5000 shillings. There are those who get headaches and the headache resulting from family planning isn’t like the normal headache that pains from here ((touching both sides of the head)), for it, it pains in the eyes and you have to buy the medicine. There are those who say they sweat a lot, every time they are feeling hot, there are problems.

I. ok, but how about at the health centre, if you came like you are suffering from malaria, do they give you medication?

R. Yes

I. how about the medicine that helps with the side effects?

R. it’s never available and when they are talking, they examine you and tell you that ‘you have this problem, but we do not have treatment for it.’ The medicine that we buy in clinics should also be here in [public] health centres. Sometimes you come without money. Me I had some money but there are times when you do not have even 100 Uganda shillings but you are bleeding.

But at the first time, I thought I was going to die. I spent a full month bleeding. I thought I was going die because whenever I was standing, I would feel my legs shaking. My husband was concerned and insisted that we should go and see the doctor. If you do not have money, do you see how you die?

………………………………………..

R. family planning wouldn’t be bad but we find problems after using it. For example me, I spent the first 5 months without getting my periods and when I got my periods, I was not stopping and when I tried to call the phone, the number they gave us on the card wasn’t available. I called and tried to call and it wasn’t available then I said inside me that maybe they deceived us because when they were teaching us, they said that whenever you get a problem, call this number it will be available and we shall give you the necessary advice on what you have to use.

When I called it and it wasn’t available, then I went to these doctors these our doctors in Nabweru health center, there is a doctor called Dr**.** XXXX, I went and he prescribed for me some small tablets which I have forgotten the name, you take I a day for 2 days. When I swallowed them, the bleeding stopped then I stopped for I month, when I stated again it was worse and now I feel very bad and I was waiting for them when they come back here, they remove it and many women have a similar problem because they come here, someone says she doesn’t go in her periods, another one say if she goes in her periods, she doesn’t stop. Now they said, they will be coming back and asking us our experiences but when they come back, they are on their programs. This makes us feel bad because they are trying to help us but when we are experiencing problems … when you are there and you are bleeding … I got a bad experience I was put on drip and put in 3 bottles, I was doing badly and my husband had said that I stop using family planning.

I. now madam, which family planning method is that, that you have been talking about, that you have been using?

R. The implant.

I. ok

R. Now you said the doctors gave you their number and sometimes you call it and it doesn’t go through, you are like saying that you don’t get any help even if you get a problem?

R. yes, there is no help because they would be helping us on the phone they give but the phone is never on. Even if I give it to you right now, it can’t go through. Now you see, they give you a free service but you end up spending because the medicine that you buy is for 3000 for a month and after a month you come back to another thing and again you buy for 3000 and after 3 years, what they had put in you has to be removed and that means getting pregnant and I don’t want to give birth anymore. I don’t want to give birth now.

I. I understand you. Now you said they put implants for free but you end up spending. Now, you have spent 9 months …

R. Yes I have spent 9 months

I. how much money have you spent?

R. I count 6000 now because when I am not well. I take the medicine because when I swallow for a month and the bleeding stops, then the next month I have to swallow. I am like someone with AIDS.

I. Are you still taking the medicine?

R. Yes. I am on medicine because you can’t be with a man and every time you are saying that you are bleeding. He will get disgusted. Men aren’t patient, he will ask you to remove it, and they don’t care whether you get pregnant or not. I have not told my husband that I have bought the tablets again. The first time he is the one who bought them because the doctor asked him to buy them and he thought that I got well. Now I went and bought them myself because if I had told him, he would have asked me to stop using contraception. So I kept it to myself.

I : What other methods have you used before?

R: Nothing. I started with the implant and that's what l have even now.

**Source transcript: IDI-FP-KS-TR-05 (40 years)**

I: You told me people use an injection because it helps them to remember when to refill and it does not burden them to have it everyday; what about an implant, what are some of the reasons that people are using it?

R: Depending on those implant users I talk to, they give them implants that can last for 3 years and others are given the one of 5 years so they use it because it takes sometime to expire, but that period is too long for me, but anyway everybody has her own preference.

I: Let us talk about you as a person, you told me you have discontinued using an injection; do you expect resuming this injection in the next few months?

R: I will first give myself sometime for some good months.

I: Why?

R: You know that injection [DMPA], it reduces your libido and in the event that you have sex with your husband, you just force it. You do not enjoy and at times it is very dry [not lubricated]. If you add the injection to the general mood swings you totally lose sexual appetite. I used to notice it. I do not know whether even those who use pills and implants feel the same

I: At some point when we were talking you told me you have ever used pills, what else have you ever used?

R: Nothing else apart from a condom.

I: What was your experience about the pills?

R: During the time I used pills I could get nausea, I could spit a lot and I discontinued it I didn’t even use it for a long time; I didn’t even complete the packet and I stared using an injection; it was less than a month.

**Source transcript: IDI-FP-NJ-TR-03 (40 years)**

I: These modern methods didn’t work well for you as a person what would you tell a friend who need advise about these modern contraception?

R: Modern contraceptives are not bad but you when you use them you need to follow its rules, when you follow the rules, they wouldn’t be bad but the problem with human being also we forget, you forget to take the pill and that very day you will conceive and you will continue to take, so that is the challenge, so people should continue to use family planning it is not bad, it is only those complications we get like over bleeding, getting nausea, getting headache because you may stop using but you will end up going back on family planning.

I: Now that you have got those challenges after using family planning, what is your opinion about these modern contraceptives?

R: In opinion, I feel want to go back for an injection.

I: Why injection yet you have an option of a condom?

R: A condom is hard you can’t use it for a full year and a year is even big, men are not reliable, he might say I am tired of a condom I don’t want it but if you go back and get an injection it is better that a condom because many men don’t have using a condom.

I: As we wind up, I would like you to tell me about anything very important that you feel we should talk about in regard to family planning that will help a Ugandan citizen?

R: There are some methods I hear only that I have never used them, I hear IUD, sterilization, and an implant.

I: What do they talk about them?

R: They say it is for five years, some people say it is also dangerous that it makes someone grow very fat and it will disappear in the body and this will make you scared, and you hear that when they insert an IUD it can enter in the uterus. They say that there are many cases of cancer in Mulago [national referral] hospital due to the IUD. So we get scared and we have failed to make choice of what method to always use

………………………………………………………

I am not using anything right now but I used some pills first then I also used an injection as I told you.

I: Why did you choose to use the pills?

R: I used the pills because I was breast feeding during that time that was the reason I was given pills. There are specific pills for mothers who breastfeed.

I: Was it your own choice to use these pills or it was given because you were breastfeeding during then?

R: I didn’t know anything much about family planning during that time, it was my first time to use family planning after I delivered my first born, the moment I started my menstruation I wanted to start on family planning; so when we went for immunization they health educated us about family planning and they told us pills are better on someone who breastfeeds so that you don’t lose breast milk.

I: Okay

R: But Musawo I didn’t know whether I was the one who made a mistake because I reached time when they even washed in my stomach, I might have made a mistake I might have missed some days to take pills. I got pregnant [while taking pills] and the foetus was damaged because I continued taking them [without knowing I was pregnant]. They had to wash my uterus because it was a pre-mature. It was so painful. I got complications and we went to the hospital. They tested me and found that I was pregnant. I was breastfeeding and at the same time I was taking some pills and that time I was feeling a lot of pain in the stomach and I was crying all night. So when we went to Mulago [national referral hospital] for a check-up, they told me I had a dead foetus. Unfortunately the health workers also didn’t explain to me what might have caused that because the pregnancy was young it was around 2 months but the pain was too much.

I: You got that challenge after how long on pills?

R: I will not deceive you musawo, what I am telling you it is not a long time because it happened immediately after producing my first born but she is now 17 years old so after my first born that is when I got that challenge but knowing it was pregnancy they first tested me in Mulago and I confirmed but I wouldn’t know if they didn’t check me; this would be my second born; then after that I produced a baby boy and he is in primary seven now, he is 14 years now, 15 years.

**…………………………………………………………………..**

I: Did you get the herbs form the health facilities?

R: No, I used to get it from friends, they used to tell me you can you these since I had hated the modern contraceptives because of the complication I got from the pills but you never know it was my mistake because by that time I was still young and I couldn’t know what would come next and I continued to take but now that I am mature I now know everything.

I: For how long did you take using the herbs?

R: It wasn’t a long period because if the boy is 15 years and the girl is 17 years the period was not so long it was like two years.

I: How did you get that pregnancy yet you were taking some herbs?

R: After stopping using pills, I used some local methods. I had something I was using, it was a thread and I had tied it in my waist so when I lost that thread that is when I got pregnant after looking for it and I failed to find it but I had stayed for sometime without getting pregnant when I was using that thread so that is when I got that pregnancy.

**Source transcript: IDI_FP_KS_02_SA (35 years)**

M: What are the most popular modern methods of contraception in this community?

R: The women I talk to most of the time use different methods of family planning but still most of them inject.

M: Now let’s talk about you own life .What method of contraception are you currently using

R : Am currently using the IUD but I started with the pills. I later stopped them and got pregnant. I had taken them for three years but people say when you swallow them they settle in one specific place in the stomach. Just imagine how big the ball in my stomach would be by now! I also hear they cause fibroids, so I had to change. I started using the implant after the birth of my baby though it treated me very badly compared to the pills to the extent that I felt like dying .I became so slim and had to go back and get it removed .So after that we were called to go for a seminar about family planning where we were told that the IUD is good because it has no chemicals in it at all and therefore it is better than any other method that contains chemicals like the pills, implant and injections, so for that I decided to use the IUD.I have so far spent 1 year using the IUD.

M: How do you like the family planning method that you are using?

R: The good thing with it is that you are not under pressure to take it daily like for the case of pills and going back after 3 months like for the injection. But what my ,is that will I not contract cancer but even I want t o go to mulago and test for cancer because I do feel something abnormal in my stomach. Basically I have no problem with this IUD a part from the fear of contracting cancer of the cervix.

**Source Transcript: IDI_FP_WK_01_SA (26 years)**

M: Why did you decide to use that injection ?

R: Ok for me they did a blood test and told me that the injection would work for me well so that is why I decided to use it too.

From the start I have been on the injector plan up to now.

M: For how long have you used this method?

R:From the time I gave birth to my baby , I have spent 7 years and 6 months using it up to now.

M:Have you ever stopped using that injection?

R: I will not lie to you. From the time I started using it, I had never stopped using it, whenever they would give me dates to return I would return on these dates.

M: How old were you when you started this method?

R: By the time I started this injection , my first born baby , I gave birth to it when I was 20 years old after its birth when it was 3 months I came to consult the health workers about family planning and I started the injection immediately , the I stopped the injection when my baby was four years old, after that I got pregnant , after that I gave birth to a baby boy when he became 4 months old I came back and started the injection again up to date.

So I have never used any other method in my life apart from the injection.

M: How do you like the family planning method that you are using?

R: For me in general I do not have bad things associated with it that I have found because I have been following the instructions well. I have only had prolonged monthly periods and I delayed going into my periods for like 3 months. The Injection has helped me to have my children grow well. If I was not using it, I think I would produce every year, but it has helped me to space them as I wanted. Those are the good things that I have received from using the injectaplan. .

M: How do you cope with the challenges in using modern family planning methods.

R: You mean how I cope up. Like if I get a problem. I always talk to the health provider and she finds a way of helping me. I cannot take a decision on my own regarding these challenges. When I got the injection and experienced problems, I came back and talked to her. She told me ‘such things happen at the start but you will be fine after some time’, and I indeed I got well after

M: So have you ever gone back to the health provider to and asked about any challenges that you faced.

R: Yes , when I got the injection it made it made me get prolonged monthly periods so I came and talked to the provider and she told me that such things happen at the start but you will be ok so after some time I got well as she had told me.

M: Did she give you any medication?

R: She did not give me any medication , she told me to be patient and see the outcomes which I did as told and I got well without taking any medication.

**Source transcript: IDI_FP_NB_02_SA (22 years)**

M: How do you access these methods?

R: I did not know anything about family planning before I gave birth, I would just hear people talk about them, and even after birth I knew nothing since I had not gotten a chance to be told about them by a professional.

I first used an injection without the knowledge from a private clinic even the nurse told me nothing, when I told her that I wanted family planning she just injected me the injection. She just told me that she had injection and pills yet I could not take pills since I see them as normal tablets yet I hate tablets.

M: Let us talk about the people in your life, how supportive are they about using these methods of family planning?

R: They [other women friends] were telling me that the IUD changes location once inserted and goes deep into the body of the uterus and causes cancer. As a human being I am scared. Women talk a lot here. My friends discourage me because they are not using family planning. So when I hear them say this, it makes me feel bad yet I do not want to follow their negative talk

But when you talk to the provider and she explains everything to you, it is better not to take what other women tell you and base on what the provider tells you or what your husband likes. I have heard all that but still use my IUD as me.

M: Have you used any modern method in the past but have since stopped using them? IF YES, tell me about those methods.

R: Yes, I have been using injection. Women say it causes headache, you become slim but what I say is that it has no problem when it adapts to your blood, I did not get any problem using it apart from peoples words. It only causes headache in the first month before it gets adapted to the body. I do not have much to say about it.

**Source transcript: IDI-FP-NM-TR-01 (25 years)**

I: Are there some methods of contraception that you would like to use but you cannot for some reasons?

R: I would like to use some but I cannot because I don’t know some of the methods and with an implant I wanted it very much but because of the side effects I had to stop using it so I don’t have any other method that I know that I would say I could use.

I: How old were you by the time you started using family planning?

R: I was about 20 years because I had 3 children and this boy is the first one; I started using family planning on my third born.

I: Let us talk about the people in your life; how supportive are they about using these methods?

R: I didnt tell any person about my side effects with implants except the health providers because people can spread rumours and yet my husband does not want me to use it. Even when I got complications I could continue using because I never wanted to produce more children, I already have enough children yet I am still a young girl.

**Source transcript: IDI_FP_KS_04_SA (25 years)**

Basically the people are my family friends that I talk to and advise me.

M: Have you used any modern method in the past but have since stopped using them? If yes, tell me about that method?

R: Yes, I need an injection previously and used it for a period of month but got no major problem with it a part from the first that has expired it I got from private clinic before turning to gov’t facilities and I formed implants the last time I meant to take m y injection and got decided to try the implants since have been told that implants do coordinate well within the injection and there have no major problem as well, if the injection treated well then the implants would treat you well. That is it sir.

The implants were recommended to me by the health worker even though I knew a neighbour who was using them. But that woman had suffered with them so much and they were removed. But for my case, the health worker told me it would match with my body since I had used the injectables before. So I followed her recommendation to use it. And now I am using implants.

M:What would you want to tell to other men/ women who have started using contraception on those who have discontinued but are sexually active?

R: l advice all the women to use family planning since it helps you to rest producing and space your children well and you can only achieve this through the use of family planning
